# Central Actions of 3α,5α-THP Involving NMDA and GABA_A_ Receptors Regulate Affective and Sexual Behavior of Female Rats

**DOI:** 10.3389/fnbeh.2020.00011

**Published:** 2020-02-11

**Authors:** Cheryl A. Frye, Alaa Qrareya, Danielle C. Llaneza, Jason J. Paris

**Affiliations:** ^1^Department of Psychology, The University at Albany—The State University of New York (SUNY), Albany, NY, United States; ^2^Biological Sciences, The University at Albany—The State University of New York (SUNY), Albany, NY, United States; ^3^Centers for Neuroscience, The University at Albany—The State University of New York (SUNY), Albany, NY, United States; ^4^Life Sciences Research, The University at Albany—The State University of New York (SUNY), Albany, NY, United States; ^5^Department of Biomolecular Sciences, The University of Mississippi, University, MS, United States

**Keywords:** allopregnanolone, anxiety, bicuculline, dizocilpine, lordosis

## Abstract

The neurosteroid, 5α-pregnan-3α-ol-20-one (known as “allopregnanolone” or 3α,5α-THP), is produced in the midbrain ventral tegmental area (VTA), independent of peripheral sources of progestogens, where it has potential actions at N-methyl-D-aspartate (NMDA) and GABA_A_ receptors to facilitate rodent sexual behavior. Progestogens can also have anti-anxiety effects, but whether these involve actions of centrally-derived 3α,5α-THP or these receptors to support reproductively-relevant behavior is not well understood. We investigated the extent to which 3α,5α-THP’s actions *via* NMDA and/or GABA_A_ receptors in the midbrain VTA influence reproductive behaviors. Estradiol-primed, ovariectomized/adrenalectomized (OVX/ADX) rats received midbrain VTA infusions of vehicle, an NMDA receptor blocker (MK-801; 200 ng), or a GABA_A_ receptor blocker (bicuculline; 100 ng) followed by a second infusion of vehicle or 3α,5α-THP (100 ng). Reproductively-relevant behaviors were assessed: sexual (paced mating), anxiety-like (elevated plus maze), and social (partner preference, social interaction) behavior. Compared to vehicle, intra-VTA infusions of MK-801 exerted anxiolytic-like effects on elevated plus maze behavior and enhanced lordosis. Unlike prior observations in gonadally-intact rats, intra-VTA bicuculline had no effect on the behavior of OVX/ADX rats (likely due to a floor effect). Subsequent infusions of 3α,5α-THP reversed effects on lordosis and infusions of bicuculline inhibited 3α,5α-THP-facilitated lordosis. Thus, NMDA and GABA_A_ receptors may act as mediators for reproductive behavioral effects of 3α,5α-THP in the midbrain VTA.

## Introduction

Progesterone (P_4_) plays a key role in the regulation of reproductive behavior in female rodents. In the brain, P_4_ can exerts its effects either *via* “genomic” or “non-genomic” action. In the hypothalamus, P_4_ facilitates lordosis, the reflexive posture that allows copulation. In this brain region, P_4_ actions are mediated by intracellular cognate progestin receptors, which act as nuclear transcription factors to alter RNA transcription and protein synthesis (Meisel and Pfaff, 1985). However, In the midbrain ventral tegmental area (VTA), P_4_ can exert actions to mediate both “consummatory” reproductive behavior (e.g., lordosis) and “appetitive” reproductive behaviors (e.g., proceptivity indicators such as ear wiggling, hopping, darting, and inhibition of normative anxiety-like behavior) which are etiologically important for successful reproduction. Given that the VTA is largely devoid of progestin receptors, P_4_ actions in this brain region are mediated by conversion of P_4_ to its 3α-hydroxy, 5α-reduced metabolite, 5α-pregnan-3α-ol-20-one (known as “allopregnanolone” or 3α,5α-THP). Unlike P_4_, 3α,5α-THP lacks affinity for progestin receptors and instead acts at neurotransmitter receptors. We have found that engaging in a pseudo-naturalistic mating paradigm (termed “paced mating”) enhances steroidogenesis of 3α,5α-THP in the midbrain and additional brain regions (hippocampus, prefrontal cortex, and diencephalon; Frye et al., 2007). Blocking 3α,5α-THP formation in the VTA increases anxiety-like behavior and attenuates lordosis of female rats or hamsters (Frye and Vongher, 2001; Petralia et al., 2005; Frye et al., 2008a,b, 2009a,b; Frye and Paris, 2011). Thus, 3α,5α-THP actions in the VTA are necessary for the full expression of consummatory and appetitive reproductive behavior. However, the mechanisms of 3α,5α-THP action in this brain region are not well-understood.

One mechanism by which 3α,5α-THP may alter reproductive behavior occurs *via* actions at inhibitory and excitatory neurotransmitter receptors. A well-characterized, non-genomic signaling pathway by which 3α,5α-THP acts is *via* modulation of inhibitory γ-aminobutyric acid type A (GABA_A_) receptor complexes. 3α,5α-THP is among the most potent, positive allosteric modulators of GABA_A_ Cl^−^ channels and a direct agonist in high concentrations (Majewska et al., 1986; Lambert et al., 1987; Morrow et al., 1987; Paul and Purdy, 1992; Gunn et al., 2011). Pharmacologically-enhancing or -attenuating actions at GABA_A_ receptors in the VTA has commensurate effects to enhance or attenuate P_4_-facilitated lordosis (DeBold and Frye, 1994). Less well understood are 3α,5α-THP’s actions that may involve excitatory N-methyl-D-aspartate (NMDA) receptors. While free 3α,5α-THP has little affinity for NMDA receptors (Maurice et al., 2006), when sulfated it acts as a negative allosteric modulator of NMDA Ca^2+^ channels, binding the NR2B subunit (Johansson and Le Grevès, 2005). Blocking NMDA receptors in the VTA also enhances progestogen-facilitated lordosis in gonadally-intact, or ovariectomized, rats (Petralia et al., 2007; Frye and Paris, 2011), but it is not known if these effects involve upstream actions of 3α,5α-THP.

Beyond lordosis, progestogens’ actions at GABA_A_ and NMDA receptors in the midbrain VTA may also be important for the expression of additional reproductively-relevant behaviors such as anxiety. Indeed, 3α,5α-THP exerts robust anti-anxiety effects in the VTA (Frye et al., 2006a) and GABA_A_ receptor agonists in the VTA can underlie positive changes in mood and affect (Gifkins et al., 2002; Genud et al., 2009). Less is known about the role that NMDA receptors may play in the VTA; albeit, NMDA receptor antagonism facilitates lordosis and may underlie aspects of anxiolysis, in part, *via* actions of peripheral glands (adrenals and/or ovaries) and/or neurosteroidogenesis (Frye and Paris, 2011). Notably, the NMDA receptor antagonist, MK-801 (a.k.a. dizocilpine), blocks 3α,5α-THP’s anti-depressant-like effects in the amygdala of rats (Shirayama et al., 2011) supporting a modulatory role for affective behavior. We have previously utilized ovariectomized (OVX) and/or adrenalectomized (ADX) rats to reveal that central neurosteroid enhancement in the VTA is important for the expression of consummatory (i.e., lordosis) and appetitive (i.e., social and anti-anxiety-like) behavior. Antagonizing GABA_A_ and NMDA receptors within the VTA alters the anxiety-like and lordosis response to pharmacologically-promoted increases in steroidogenesis (Frye and Paris, 2011), but the identity of the important steroids that underlie actions at these sites are not known. The present work aimed to assess the importance of GABA_A_ and NMDA receptor targets in the mediation of 3α,5α-THP’s central effects to influence reproductively-relevant behavior. We hypothesized that female OVX/ADX, estradiol (E_2_)-primed rats administered the NMDA receptor blocker, MK-801, would demonstrate reduced anxiety-like behavior (general anxiety assessed *via* the elevated plus maze and social anxiety assessed *via* a social interaction test) and enhanced sexual receptivity (assessed *via* a propinquity test and a paced mating test), while those administered the GABA_A_ receptor blocker, bicuculline, to the VTA, would demonstrate opposite effects on these behaviors. We further hypothesized that subsequent 3α,5α-THP infusions to the VTA would reverse the effects of blockers.

## Materials and Methods

These methods were approved by the Institutional Animal Care and Use Committee at The University at Albany-SUNY and were conducted in accordance with ethical guidelines defined by the National Institutes of Health (NIH Publication No. 85-23).

### Animals

Adult (50–60 days old), Long-Evans female rats (*N* = 100) were bred in the Life Sciences Laboratory Animal Care Facility at The University at Albany-SUNY (original stock obtained from Charles River, Raleigh, NC, USA). Rats were housed in polycarbonate cages with woodchip bedding (45 × 24 × 21 cm) in a temperature-controlled room (21 ± 1°C) and were maintained on a 12:12 h reversed light cycle (lights off at 08:00 h) with continuous access to Purina Rat Chow and tap water in their home cages.

### Surgical Protocol

Rats were stereotaxically-implanted with bilateral guide cannulae aimed over the medial aspect of the VTA (from bregma: AP = −5.3, ML = ± 0.4, DV = −7.0) under xylazine (12 mg/kg) and ketamine (70 mg/kg) anesthesia. Immediately following stereotaxic surgery, rats were OVX/ADX as previously described (Frye and Paris, 2011). Following surgery and prior to testing, animals were monitored for loss of weight, righting response, flank stimulation response, and/or muscle tone (Marshall and Teitelbaum, 1974). One rat failed these assessments and was immediately euthanized. All rats were screened for complete-ADX *via* *post hoc* assessment of corticosterone in plasma (methods below). All the rats included in analyses had circulating concentrations of corticosterone that were below baseline levels (<1 μg/dl). Sixteen rats were excluded due to circulating corticosterone >1 μg/dl, which prior work suggests is indicative of incomplete adrenalectomy and can alter behavioral and endocrine measures (Frye and Paris, 2011). Remaining rats all had circulating corticosterone levels <1 μg/dl.

### Preparation of Pharmacological Blockers

The NMDA receptor blocker, MK-801 hydrogen maleate (Sigma Chemical Co., St. Louis, MO, USA) was diluted to a concentration of 200 ng/μl in sterile saline as elucidated in prior investigations (Frye and Paris, 2011). MK-801 is a long-lasting non-competitive antagonist that acts in the receptor channel pore, where it blocks opening (Dravid et al., 2007). While, MK-801 and 3α,5α-THP have not previously been co-infused in a mating model, this dose has previously been demonstrated to facilitate reproductive behaviors and antagonize the effects of a general neurosteroidogenesis enhancer (Petralia et al., 2007; Frye and Paris, 2011).

The GABA_A_ blocker, bicuculline (Sigma Chemical Co., St. Louis, MO, USA) was dissolved in sterile saline to a concentration of 100 ng/μl as previously demonstrated (Frye and Paris, 2011). Bicuculline is an antagonist that inhibits the GABA_A_ ion channel by competing for the GABA binding site on GABA_A_ receptors but does not compete with the allosteric steroid-binding site (Ueno et al., 1997). This dose has previously been demonstrated to reduce anxiety-like behavior and lordosis of sexually-receptive rodents (Frye et al., 2006b; Frye and Paris, 2009).

### Study Procedure

**Figure 1:**
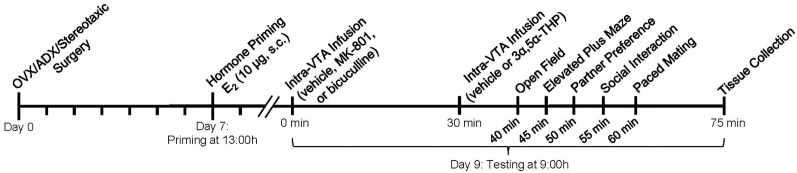
Time-course of the experimental procedure.

Seven days after surgery, OVX/ADX rats were primed with E_2_ (10 μg, SC) and tested 44 h later to evaluate sexual behavior (Figure 1). On the day of testing, E_2_-primed rats were randomly assigned to receive a bilateral infusion (1 μl) of saline vehicle, bicuculline (100 ng/μl), or MK-801 (200 ng/μl) to the midbrain VTA. Thirty min later, rats received a subsequent infusion of 25% β-cyclodextrin vehicle or 3α,5α-THP (100 ng/μl). Rats were tested 10 min later in all tasks described below (Figure 1). We have previously systematically assessed the effects of exposure to the tasks utilized in this behavioral battery (Frye et al., 2007). We find that performance in one task does not significantly influence subsequent behavioral performance or central/circulating steroid levels, with the exception of an engagement in paced mating which promotes central steroidogenesis of 3α,5α-THP (Frye et al., 2007). As such, the paced mating task is always performed last (Figure 1). There were six experimental conditions based on intra-VTA infusions: vehicle/vehicle (*n* = 13), vehicle/3α,5α-THP (*n* = 17), MK-801/vehicle (*n* = 15), MK-801/3α,5α-THP (*n* = 12), bicuculline/vehicle (*n* = 15), and bicuculline/3α,5α-THP (*n* = 11). The numbers of rats per group were reduced when those with infusions to sites other than the VTA were taken into account.

### Behavioral Outcome Measures

Behavioral data were collected using ANY-maze animal tracking software (Stoelting Co., Wood Dale, IL, USA). All rats were placed in an open field apparatus (76 × 57 × 35 cm) consisting of a 48-square grid floor (6 × 8 squares, 9.5 cm/side) for a 5 min habituation to the apparatus and testing room. The frequency of crossings into each of the 48 squares on the floor was recorded as an assessment of general motor behavior. While entries into the central 24 squares of the open field is a common measurement of anti-anxiety behavior (Frye and Rhodes, 2006a), we observed no differences in the number of central squares entered among experimental groups in this study, or prior studies utilizing this OVX/ADX model (Frye and Paris, 2011).

#### Elevated Plus Maze

The elevated plus maze was conducted per previous methods (File, 1990). Briefly, the maze has four opaque arms (49 cm long, 10 cm wide) elevated off the ground (50 cm high). Two arms (east and west) are enclosed by walls (30 cm high), while the other two arms (north and south) are exposed. The amount of time spent on, and the number of entries into, open or closed arms were recorded during the 5 min task. Open arm time is an index of exploratory and anti-anxiety behavior, while total arm entries are an index of motor behavior.

#### Partner Preference

Partner preference was conducted as previously described (Frye and Rhodes, 2006a). Briefly, stimulus rats (one diestrous female and one male) are confined to opposite corners of an open field *via* Plexiglass compartments that are permeated with small holes for olfactory exchange. Sexually-receptive rats will seek an opposite-sex partner for the purposes of copulation (Nofrey et al., 2008). The amount of time that an experimental rat spends in proximity (one body length or less) to either stimulus rat is recorded during a 5 min test. In the lab setting, preference for a male vs. female stimulus rat is considered a measure of sexual receptivity.

#### Social Interaction

Social interaction was assessed in the open field apparatus per previous methods (File, 1990). Briefly, an experimental female was placed in one corner, while a diestrous stimulus female was placed in the opposite corner of the apparatus. The amount of time that the experimental rat spent interacting (sniffing, crawling over or under, following with contact, tumbling, boxing, or grooming) with the stimulus rats was recorded in a 5 min test. Total time spent in social interaction is a measure of social anxiety-like behavior.

#### Paced Mating

Paced mating was conducted per previous methods (Erskine, 1985). In brief, the paced mating apparatus (37.5 × 75 × 30 cm) was equally divided by a Plexiglas partition, which contained a small (5 cm in diameter) hole in the bottom center, allowing the female (but not the stimulus male) free access to both sides of the apparatus. Frequency of mounts + intromissions + ejaculations was recorded and a lordosis quotient was calculated [(frequency of female dorsiflexion during a sexual contact/total sexual contacts by a male) * 100] during a 15 min test. As well, the percentage of aggressive behaviors (vocalizing, attack) and the percentage of times the experimental female left the chamber containing the male (% exits) following sexual contacts were recorded.

#### Cannulae Placement and Neuroendocrine Assessment

Immediately after testing, rats were decapitated and trunk blood and whole brains were collected and stored as described (Frye and Rhodes, 2006a). Infusion site analyses were conducted on fresh tissue, as described (Frye and Paris, 2011). Of the rats that had not been excluded for receiving a partial-ADX, 22 had infusions to sites other than the VTA; however, all experimental groups were not represented, which precluded factorial analyses of behavioral measures based on-site placement. As such, rats with cannulae placement incongruous with a hit to the VTA were excluded from all analyses. Thus, the experimental groups with complete-ADX and verified cannulae placement to the VTA yielded: vehicle/vehicle (*n* = 9), vehicle/3α,5α-THP (*n* = 11), MK-801/vehicle (*n* = 9), MK-801/3α,5α-THP (*n* = 12), bicuculline/vehicle (*n* = 11), and bicuculline/3α,5α-THP (*n* = 9). Plasma was extracted for radioimmunoassay of corticosterone.

#### Steroid Extraction

Corticosterone was extracted from serum *via* incubation with ether and 800 cpm of [^3^H] corticosterone (Frye and Bayon, 1999). Ether-incubated steroids were snap-frozen twice and supernatant was evaporated in a speed drier. Samples were reconstituted with phosphate assay buffer to the original serum volume (Frye et al., 2008a,b).

#### Radioimmunoassay

Levels of corticosterone were measured by radioimmunoassay, per previously reported methods (Frye et al., 1998). Concentrations of [^3^H] corticosterone were assessed *via* the logit-log method of Rodbard and Hutt (1974) with “AssayZap” interpolation software published by Biosoft (1994). Inter- and intra-assay reliability coefficients were 0.04 and 0.07, respectively.

### Statistical Analyses

Data were analyzed using StatView (SAS Institute Inc.). Group differences were assessed *via* two-way analyses of variance (ANOVAs) with central blocker condition (vehicle, MK-801, bicuculline) or central 3α,5α-THP condition (vehicle, 3α,5α-THP) as factors. Significant main effects were followed by Fisher’s protected least significant differences *post hoc* tests to determine group differences. Significant interactions were delineated *via* follow-up one-way ANOVA with alpha corrected for all possible comparisons. The alpha-level for statistical significance was *p* < 0.05.

## Results

### MK-801 and 3α,5α-THP Infusions to the VTA Altered Anti-anxiety Behavior

**Figure 2:**
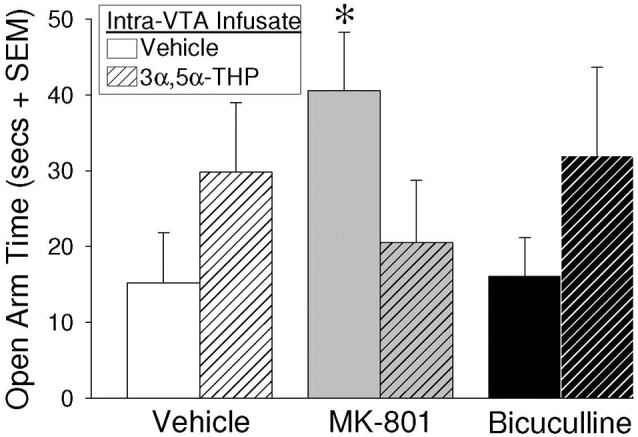
Depicts time spent on the open arms of an elevated plus-maze among estradiol-primed (10 μg, SC), ovariectomized/adrenalectomized (OVX/ADX) female rats infused with the vehicle, MK-801, or bicuculline, followed by subsequent infusions of vehicle or 3α,5α-THP, to the ventral tegmental area (VTA) of the midbrain. *Indicates significantly different from vehicle/vehicle- or bicuculline/vehicle-infused rats, *p* < 0.05.

Two-way ANOVAs revealed that intra-VTA infusion of blockers and 3α,5α-THP significantly interacted to influence open arm time in the elevated plus-maze (*F*_(2,55)_ = 3.05, *p* = 0.05; Figure 2). Contrasts revealed that OVX/ADX rats infused with MK-801/vehicle spent a significantly increased amount of time on the open arms of the elevated plus-maze, compared to rats receiving infusions of bicuculline/vehicle (*p* = 0.04) or control infusions of vehicle/vehicle (*p* = 0.046). Rats infused with subsequent 3α,5α-THP did not significantly differ from vehicle-infused controls or any other group. Notably, central manipulations did not significantly alter motor behavior in the open field (indicated by the number of total squares entered) or in the elevated plus-maze (indicated by the number of arms entered; Table 1).

**Table 1:** Motor and social behavior measures of female ovariectomized/adrenalectomized rats infused with the vehicle, 3α,5α-THP, bicuculline, and/or MK-801 to the ventral tegmental area of the midbrain (mean ± SEM).

Infusate #1	Vehicle		MK-801		Bicuculline	
Infusate #2	Vehicle (*n* = 9)	3α,5α-THP (*n* = 11)	Vehicle (*n* = 9)	3α,5α-THP (*n* = 12)	Vehicle (*n* = 11)	3α,5α-THP (*n* = 9)
*Open field*
Number of total entries	265 ± 20	273 ± 18	259 ± 22	270 ± 21	274 ± 21	254 ± 25
*Elevated plus maze*
Number of total arm entries	8 ± 1	11 ± 2	12 ± 2	10 ± 2	9 ± 2	11 ± 3
*Partner preference*
Time with male (s)	159 ± 14	152 ± 18	133 ± 33	138 ± 27	120 ± 25	171 ± 26
Time with female (s)	90 ± 12	96 ± 19	107 ± 30	91 ± 28	95 ± 21	72 ± 16
*Social interaction*
Interaction time (s)	84 ± 15	87 ± 10*	132 ± 12	84 ± 16*	99 ± 10	74 ± 14*
*Paced mating*
Pacing exits (%)	19 ± 6	15 ± 5	11 ± 5	12 ± 6	11 ± 5	6 ± 4

*Indicates significant main effect for the performance of rats receiving subsequent infusions of 3α,5α-THP to differ from those that received subsequent infusions vehicle, irrespective of additional MK-801 or bicuculline treatment, *p* < 0.05.

### 3α,5α-THP Influenced Non-sexual Social Behavior

Central manipulations also altered non-sexual social behavior. There was a main effect for subsequent 3α,5α-THP infusions to decrease the duration of time spent in social interaction with a conspecific (*F*_(1,55)_ = 4.65, *p* < 0.05); this was observed irrespective of whether vehicle, MK-801, or bicuculline was first infused (Table 1). While, it was apparent that this effect was observed only in MK-801 and bicuculline-infused groups when 3α,5α-THP was co-administered, these factors did not significantly interact.

In the partner preference task, neither infusions of central pharmacological blockers (MK-801, bicuculline) nor infusions of subsequent 3α,5α-THP, significantly influenced the number of time rats spent in proximity to a male (Table 1).

### MK-801 and 3α,5α-THP Facilitated Lordosis and Defensive Aggression Behavior in Response to Mounting

**Figure 3:**
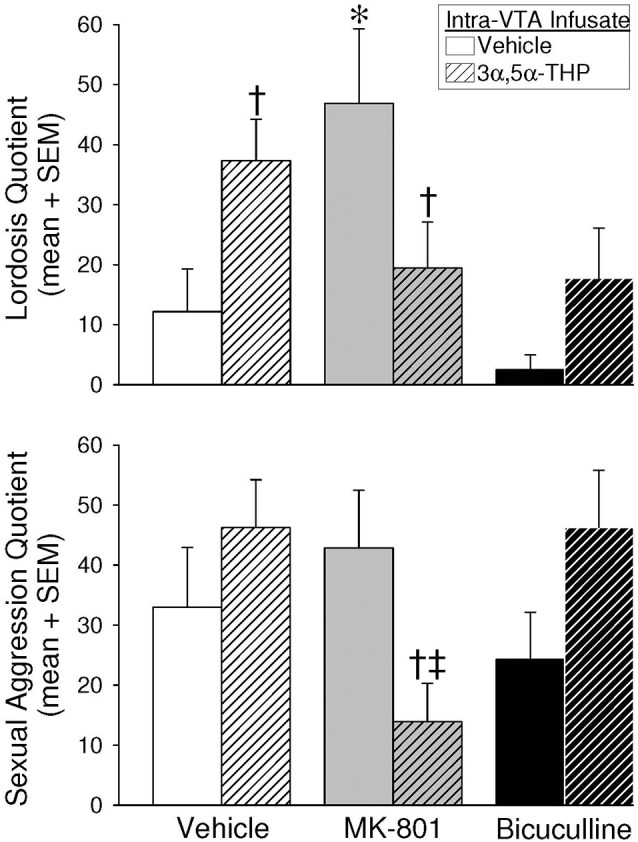
Depicts lordosis (top) and defensive sexual aggression (bottom) quotients in the paced mating paradigm among estradiol-primed (10 μg, SC), OVX/ADX female rats infused with vehicle, MK-801, or bicuculline, followed by subsequent infusions of vehicle or 3α,5α-THP, to the VTA of the midbrain. *Indicates significantly different from vehicle/vehicle-infused controls. ^†^Indicates a significant difference between rats receiving subsequent 3α,5α-THP infusions compared to their respective vehicle/vehicle or MK-801/vehicle infused controls. ^‡^Indicates significantly different from vehicle/3α,5α-THP or bicuculline/3α,5α-THP-infused rats, *p* < 0.05.

Intra-VTA infusions of blockers and 3α,5α-THP significantly interacted to alter lordosis (*F*_(2,55)_ = 6.54, *p* < 0.05; Figure 3, top). Infusions of vehicle/3α,5α-THP significantly enhanced lordosis compared to vehicle/vehicle-infused controls (*p* = 0.03) or bicuculline/vehicle-infused rats (*p* = 0.002). MK-801/vehicle also significantly enhanced lordosis compared to vehicle/vehicle controls (*p* = 0.004), or rats infused with MK801/3α,5α-THP (*p* = 0.01), bicuculline/vehicle (*p* = 0.0002), or bicuculline/3α,5α-THP (*p* = 0.02). Thus, infusions 3α,5α-THP or MK-801 with vehicle significantly enhanced lordosis, but 3α,5α-THP attenuated MK-801’s effects.

Defensive aggression in response to mounting was also significantly altered by intra-VTA infusions (Figure 3, bottom). Blockers and 3α,5α-THP interacted (*F*_(2,55)_ = 5.22, *p* < 0.05), such that MK-801/3α,5α-THP infusions attenuated defensive aggression compared to MK-801/vehicle infusions (*p* = 0.02), or infusions of any other compound co-administered with 3α,5α-THP (vehicle/3α,5α-THP, *p* = 0.006; bicuculline/3α,5α-THP, *p* = 0.008). The pacing of mating contacts, following mounting by males, was reduced when blockers were infused to the VTA compared to vehicle infusions; however, this was not a statistically significant difference (Table 1). Thus, the co-infusion of MK-801 and 3α,5α-THP to the VTA reduced defensive aggression compared to other manipulations.

## Discussion

The hypothesis that intra-VTA infusions of MK-801 would enhance sexual and reproductively-relevant (exploratory, affective, social) behaviors, and bicuculline would reduce these behaviors, was partly upheld. E_2_-primed, OVX/ADX rats that were infused with MK-801 demonstrated significantly enhanced lordosis in the paced mating task, and significant anxiolysis as assessed *via* the elevated plus-maze, compared to bicuculline- or vehicle -infused controls. Similarly, investigations in mouse models have revealed that systemic administration of MK-801 has commensurate anxiolytic effects to those of 3α,5α-THP (Reddy and Kulkarni, 1997) and genetically perturbing global NMDA receptor expression yields an aberrant sexual, social, and anxiety-like phenotype (Mohn et al., 1999). However, MK-801 infusions in the present study did not significantly alter non-sexual social behavior (partner preference or free social interaction). Alternatively, infusions of bicuculline significantly blocked the effects of 3α,5α-THP to enhance lordosis, but did not significantly reduce sexual, social or affective behaviors on their own. These findings are commensurate with those of past observations wherein sexually-receptive rodents that were gonadally-intact or OVX (and/or ADX and hormone primed with E_2_ and P_4_) demonstrated enhanced lordosis when NMDA receptors were blocked in the VTA (Petralia et al., 2007; Frye et al., 2008a,b; Frye and Paris, 2011) and reduced lordosis when bicuculline was infused to the midbrain VTA or central gray (McCarthy et al., 1991; Frye and Paris, 2009, 2011). These effects are dampened when peripheral steroid glands (ovaries and adrenals) are completely extirpated such that sexual and anxiety-like behavior of rats is greater in gonadally-intact > OVX > OVX/ADX rats (Fernández-Guasti et al., 1991; Gorzalka and Moe, 1994; Frye and Paris, 2009, 2011). E_2_-priming alone may not sufficiently reinstate anti-anxiety and lordosis in the OVX/ADX model to a level that intra-VTA bicuculline can efficaciously be observed to attenuate these behaviors (Frye and Paris, 2011). Indeed, circulatory P_4_ is a critical factor for several aspects of paced mating including its reinforcing properties (Paredes and Alonso, 1997; Paredes and Vazquez, 1999; González-Flores et al., 2004). For these reasons, we may have observed a “floor effect” on anxiety-like responding with exogenous 3α,5α-THP non-significantly increasing anti-anxiety-like behavior when administered alone. Thus, NMDA and/or GABA_A_ receptors in the midbrain VTA regulate lordosis and anti-anxiety-like behavior of rats; but, appetitive sexual behaviors (e.g., ear wiggling, pacing) and social interactions may require circulatory progestogens for assessment of full expression.

The second hypothesis, that subsequent 3α,5α-THP infusions to the VTA would reverse effects of pharmacological blockers, was partly supported. Enhancements of anxiolysis and lordosis that were promoted by MK-801 infusions were not observed when 3α,5α-THP was co-administered. Co-infusion of MK-801 and 3α,5α-THP also reduced defensive aggression, but this was not observed when either compound was infused with the vehicle. These data support the notion that 3α,5α-THP may play an important reproductive regulatory role *via* intra-VTA NMDA receptors. Similarly, others have seen intracerebroventricular infusions of an NMDA receptor antagonist to block P_4_-enhancement of lordosis among OVX, E_2_-primed rats (Gargiulo et al., 1992; Gargiulo and Donoso, 1995). We also observed 3α,5α-THP-mediated lordosis to be significantly attenuated when bicuculline was co-infused, supporting a regulatory role for 3α,5α-THP at intra-VTA GABA_A_ receptors. Indeed, 3α,5α-THP has been observed to regulate GABA_A_ subunit expression *in vitro* and *ex vivo* (Shen et al., 2005; Zhou and Smith, 2007) and orally-active micronized P_4_, which can metabolize to 3α,5α-THP, is seen to enhance the positive and negative effects of benzodiazepines in premenopausal women (Babalonis et al., 2011a,b). In the present animal model, it is known that neither gonadal nor adrenal, P_4_ are necessary for the expression of lordosis (Foreman and Moss, 1977; Auger et al., 1997); rather, central neurosteroidogenesis in the VTA is critical for expression and maintenance of this behavior (Frye and Paris, 2011). The present investigation extends these findings to reveal 3α,5α-THP as the important neurosteroid product acting in the VTA to mediate reproductively-relevant anxiety and sexual behavior of female rats.

We have previously observed 3α,5α-THP infusions to the VTA to be associated with enhanced 3α,5α-THP production in other brain regions (hippocampus, prefrontal cortex, diencephalon; Frye and Rhodes, 2006a). These effects are not thought to be due to infusate diffusion given that we have previously observed central infusate to spread ~1 mm and for intra-VTA infusions not to diffuse beyond the midbrain (Frye and Rhodes, 2008). Moreover, 3α,5α-THP infusions targeted to sites in proximity to the VTA (substantia nigra or central gray) are not observed to promote 3α,5α-THP formation in midbrain, hippocampus, or striatum (Frye and Rhodes, 2006b, 2008; Frye et al., 2008a,b). Rather, steroids can be synthesized in neural cells, independent of peripheral sources (King, 2008). Notably, women administered P_4_ exhibited shifts in metabolite:prohormone ratio that was indicative of depression status, supporting the importance of steroid metabolites (Girdler et al., 2012). Others find that exogenous progestins and oral contraceptives that do not metabolize 3α,5α-THP increase anxiety-like behavior of rodents (Porcu et al., 2012). Formation of 3α,5α-THP may play an important role in the benefits of pregnane steroids.

The behavioral effects of inhibitors observed herein likely involve modulation of VTA efferents to limbic and extralimbic brain regions. In the present report, we observed either 3α,5α-THP or MK-801 actions in the VTA to facilitate lordosis. The midbrain VTA consists of a mixture of dopaminergic (~65%) and non-dopaminergic neurons, the latter of which are largely GABAergic (~30%) or glutaminergic (~5%; Zessen et al., 2012). Activation of dopaminergic efferents from the VTA to forebrain structures (particularly, within the striatum) are generally observed to inhibit lordosis and lesioning or quiescing these neurons facilitates lordosis (Caggiula et al., 1979; Sirinathsinghji et al., 1986; Pednekar and Mascarenhas, 1993; Frye et al., 2010). By virtue of 3α,5α-THP’s potent affinity for GABA_A_ receptors, it rapidly promotes Cl^−^ influx into neurons, reducing excitability (Majewska et al., 1986; Lambert et al., 1987). When acting in the VTA, 3α,5α-THP may dampen the activity of dopaminergic efferents, thereby promoting lordosis. As well, intra-VTA NMDA receptors are important for dopamine neurotransmission (Gu and Lu, 2018) and infusion of MK-801 decreases Ca^2+^ influx into neurons, similarly attenuating excitation and promoting lordosis. Despite achieving their endpoints by different mechanisms, actions of 3α,5α-THP and MK-801 to inhibit dopaminergic projection neurons to other brain regions, particularly the hippocampus, mPFC, and striatum (caudate/putamen and nucleus accumbens), may underlie their effects to facilitate reproductive behavior. It is of interest that the subsequent addition of 3α,5α-THP reversed MK-801’s effects on lordosis. The capacity for 3α,5α-THP to restore behavioral homeostasis may be conferred by its capacity to potently activate GABA_A_ Cl^−^ channels, restoring ion homeostasis. Following inhibition of Ca^2+^ channels *via* MK-801, cells may become hyperpolarized; under these circumstances, subsequent activation of GABA_A_ channels *via* 3α,5α-THP may efflux, rather than influx, Cl^−^, thus, restoring the excitatory/inhibitory ion balance within the VTA.

The present study reveals the capacity for modulation of intra-VTA NMDA receptors to influence appetitive and consummatory reproductive behaviors in female rats. Subsequent administration of 3α,5α-THP restored behavioral homeostasis, presumably *via* actions at GABA_A_ receptors to re-establish ion homeostasis within the VTA. These data further reveal the importance of excitatory/inhibitory substrates within the VTA for reproductively-relevant behaviors. Formation of 3α,5α-THP may act to balance ion homeostasis within the VTA, thereby influencing efferents to regions involved in the processing of natural reward.

## Data Availability Statement

The raw data supporting the conclusions of this article will be made available by the authors, without undue reservation, to any qualified researcher.

## Ethics Statement

The animal study was reviewed and approved by the Institutional Animal Care and Use Committee at The University at Albany-SUNY and were conducted in accordance with ethical guidelines defined by the National Institutes of Health (NIH Publication No. 85-23).

## Author Contributions

CF participated in experimental design. DL and JP acquired data. CF, DL, and JP performed data analyses. CF, AQ, DL, and JP wrote and contributed to the writing of the manuscript. All authors listed, have made substantial, direct and intellectual contribution to the work, and approved it for publication.

## Conflict of Interest

The authors declare that the research was conducted in the absence of any commercial or financial relationships that could be construed as a potential conflict of interest.
